# Facile Manufacture of Oxide-Free Cu Particles Coated with Oleic Acid by Electrical Discharge Machining

**DOI:** 10.3390/mi13060969

**Published:** 2022-06-19

**Authors:** Irshad Ahamad Khilji, Siti Nadiah Binti Mohd Safee, Sunil Pathak, Chaitanya Reddy Chilakamarry, Amiril Sahab Bin Abdul Sani, Venugopal Jayarama Reddy

**Affiliations:** 1 Faculty of Manufacturing and Mechatronic Engineering Technology, Universiti Malaysia Pahang, Pekan 26600, Pahang, Malaysia; Irshad_ahamad@hotmail.com (I.A.K.); sitinadiah@ump.edu.my (S.N.B.M.S.); amril@ump.edu.my (A.S.B.A.S.); 2 Hilase Centre, Institute of Physics, Academy of Sciences of the Czech Republic, Za Radnici 828, 25241 Dolni Brezany, Czech Republic; 3 Faculty of Chemical and Process Engineering Technology, Universiti Malaysia Pahang, Gambang 26300, Pahang, Malaysia; cchaitanyareddys@gmail.com; 4 Faculty of Industrial Science and Technology, Universiti Malaysia Pahang, Gambang 26300, Pahang, Malaysia; venugopal@ump.edu.my

**Keywords:** nanoparticles, EDM, oxidation, surface finishing, coating, dielectric, oleic acid, waste

## Abstract

Particle synthesis has seen significant advances in current trends. However, the synthesis of metal particles without oxidation is a challenge for researchers. The current study presents a straightforward, convenient, and convincing approach for manufacturing copper (Cu) particles free of surface oxide. The die-sink Electrical Discharge Machine (EDM) of copper alloys with oleic acid resulted in the formation of Cu particles with diameters between 10 to 20 µm. X-ray diffraction (XRD) was used for particle examination after cleaning and sonication with distilled water. Cu particles with oleic acid coating retained a Cu phase without oxidation after synthesis. Transmission electron microscopy (TEM) and scanning electron microscopy (SEM) were used to determine the size and morphology of generated particles. Fourier transforms infrared (FT-IR) analysis revealed the oleic acid-coated Cu surface bonded with an oxygen atom. Also, the agglomeration and change of size involving Cu particles with increasing voltages in the pulse supply in EDM were reported.

## 1. Introduction

Copper particles have attracted much attention owing to their unique optical, catalytic, tribological, electrical, and thermally conductive properties [1]. In addition, copper nanoparticles are less expensive than Au and Ag. However, their oxidation hinders the practical application of Cu Nanoparticles (NPs) [2].

Copper particles are used in biomedical, electrical, mechanical engineering, automotive, chemical, petrochemical, and dental industries. The renewed interest in nanometre-sized structures (NPs) can be partly attributed to their innovative properties like enhanced surface tension, superior surface area, lubrication, adhesion, and colloidal distribution stability by flocculation [3]. The nanoparticles’ high surface-to-volume ratio and electron wavelength-scale dimensions describe their surface and mechanical effects. These bifurcations are employed for new and improved materials and cutting-edge medical, optical, and electrical technologies [4]. A nanoparticle is defined as a microparticle less than 100 nm diameter and is classified into different categories depending on the individual’s structure, shape, and size [5].

Many studies have been conducted to improve and develop techniques to obtain different materials from NPs due to the significant difference and widespread applications. The production of NPs can vary based on their use in numerous fields [6]. Engineering synthesis of NPs from different materials is achieved in different ways. The physical, chemical, biological, and hybrid processes are the four main types of synthesis, and physical processes are the most widely used. The physical processes are divided into different techniques based on the nanoparticles used. However, the working principles remain the same [7]. Many techniques involve chemicals and require much time and sophisticated technical equipment. To solve the difficulties associated with the synthesis of NPs, a technology to produce NPs by electrical discharge machining has been developed. Although the approach aims to mill the materials, the sludge/debris product was collected and evaluated as nanoparticles (NPs) [8].

**Figure 1:**
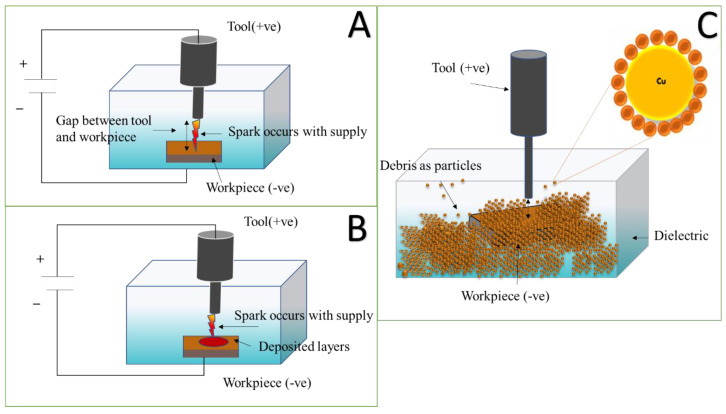
Working principle of EDM as particle synthesis. (**A**) EDM setup; (**B**) sparking process; (**C**) plasma region debris detaching in the form of particles.

The voltage applied between anode and cathode generates a temperature of about 10,000 °C during the sparking process in die-sinking electrical discharge machining (EDM). This temperature results in the formation of a plasma channel for the development of particles. Due to the localised temperature, metal evaporation in the plasma field causes erosion of the workpiece and the electrode tool, known as debris. This phenomenon is illustrated schematically in Figure 1. The continual dielectric fluid flow aids in the maintenance of a neat inter-electrode gap, and contaminants from the machining region are washed away [9]. Equipment for sports, biomedical, pharmaceutical, and motorised investigation are utilised from Electrical Discharge Machining (EDM). The EDM eliminates unwanted substances in the waste form and generates the tool’s surface structure by the recurrent electrical spark [10]. The investigation of waste debris may be highly beneficial in gaining a better knowledge of compound mechanism creation in a machine.

The workpiece serves as the anode, and the electrode serves as the cathode since it is connected to negative terminal. Kerosene, distilled H_2_O, transformer oil, and Neem oil are generally used to provide as dielectric material [11]. Free electrons from the tool are susceptible to electrostatic forces among the tool and workpiece in an electric field. Electrons will be emitted if the tool’s work function or binding energy is smaller, called cold emission for the emission of electrons [12]. The ‘cold emitted’ electrons travel by the dielectric fluid and reach the workpiece at high speeds. After gaining momentum, the electrons collide with dielectric molecules and move towards the workpiece. Depending on the electron’s work function and energy, the ionisation of the dielectric molecule may be affected [13]. Collisions between positive ions and electrons result in additional electrons being accelerated. The concentration of electrons and ions in the dielectric medium between tool and workpiece at the spark gap rise due to this cyclic action. The plasma in these channels has a high concentration, which is less electrically resistive than other channels. An avalanche of electrons occurs due to the movement of electrons and ions between the tool and the workpiece [14].

**Figure 2:**
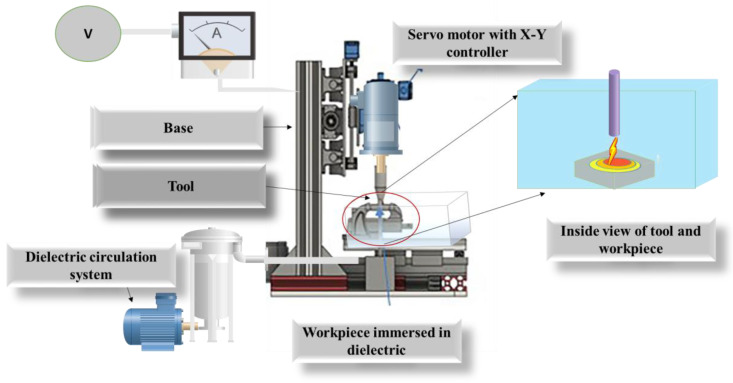
Schematic of EDM machine used for copper particle synthesis with filtration system setup.

**Figure 3:**
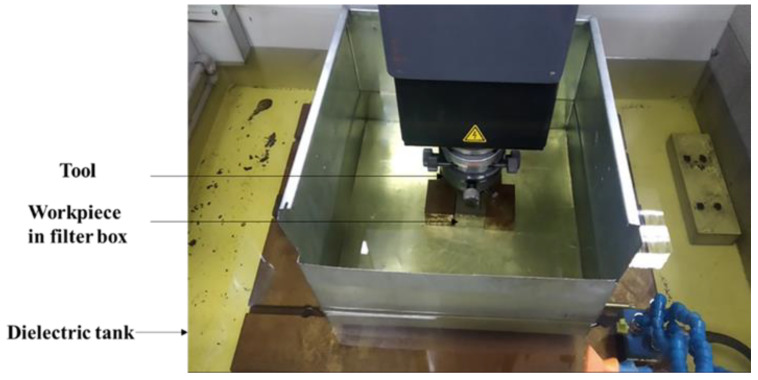
Workpiece emerged with an oleic acid layer.

The visual effect of such electron and ion moment results in a spark. This spark is dissipated as thermal energy. Negatively charged electrons emit from the workpiece, while positive ions emit from tool. The kinetic energy of ions and electrons’ affects the workpiece’s surface, causing the tool to generate thermal energy. The temperature rises to 10,000 °C in milliseconds due to the strong localised heat flow [15]. When the temperature reaches an extreme level in one area, it vaporises the material and removes some melting metals in the form of particles. EDM removes materials by creating shock waves when plasma channels collapse by discontinuous potential differences. Extreme temperature melting and material removal are caused by electrons striking the workpiece from craters. Pulse voltage fluctuates with the duration of each pulse [16]. The arc produces a targeted material elimination at a certain point, as a schematic of die-sinking EDM shown in Figure 2 and Figure 3.

On the other hand, sparks are evenly dispersed throughout the tool’s surface, resulting in a more even distribution of material removal. As a result, EDM can be used in conjunction with other machining processes to produce more debris, leading to a faster rate of alloy production. In addition, almost spherical particles made up a more extensive sample percentage, whereas elliptical form particles were smaller [17].

Several studies have reported small-sized particles known as EDM debris. Khanra et al. examined the impact of energy and particle composition in steel EDM with a ZrB_2_-Cu tool and kerosene. The debris characterisation found two types of particles: spherical and non-spherical. Workpiece and tool surfaces are sufficiently heated to produce ZrC [18]. The low energy electric discharge particles by utilising ultrashort pulse were described by Roy et al. Lesser spherical particles were mixed with a larger quantity of irregular particles at a lower voltage. Most of the spherical particles were observed in one phase, whereas the irregular particles were random [19]. Abdul Kareem et al. found that process setting and cooling approaches significantly impacted the formation of particles and the wear of electrodes [20]. Ayers et al. examined the structure and dimension of EDM debris by scanning electron microscope. They used a prospective spark erosion technique to produce the metal carbide powder and stated pulse frequency and polarity impact particle size. Powder particles ranged in size from 1 µm to 40 µm. They discovered that the bigger particles had straight polarity. EDM has been used to produce a variety of crystalline powders [21].

Walter et al. provided a new process to manufacture 90% amorphous powder with 5 nm to 75 µm, which is impossible to produce using current atomisation or melt spinning procedures [22]. Compared to traditional EDM, Shrivastava et al. found that cryogenic cooling improves and maintains the particles’ shape. The high rate of debris creation was observed by researchers who have combined the EDM process with other machining processes [23]. Soni et al. discovered that electrode workpiece and dielectric medium impacted the EDM process performance and generated particles of varying structure and size [24].

However, the drawback of EDM results in the particle with oxidation in kerosene or distilled water in the machining process and the agglomeration of particles rising with an increasing temperature of workpiece and tool. The electrical discharge method developed a simple method for synthesising Cu particles with surface free oxide. In the current research, EDM was carried out, and oleic acid-containing hexane was applied as a capping substance to obtain hydrophobic nature. The machining process of particles was followed by stirring and sonication.

## 2. Material and Method

Copper work and tool piece was obtained from Orioner high-tech sdn. Bhd., Malaysia. The workpiece’s dimensions were 30 × 50 mm, and it was made of copper. An illustration of the schematic diagram is shown in Figure 2. The research employed a copper tool with a 10 mm diameter and length of 50 mm. Copper is a soft material that requires less voltage during machining. Copper is used as a tool due to its excellent thermal and electrical conductivity, low cost, and wide availability. However, copper is highly oxidised in the normal environment compared with other conductive materials, and its crystalline structure is face-centred cubic [25]. Table 1 show experimental setup and operating and applied voltage. The “Mitsubishi E12D” Tokyo, Japan make electrical discharge die-sinking machine was used to synthesize particles. The approach used here is based on the earlier work of [8].

Oxidation-free surface copper particles were synthesised in a single step utilising a EDM explosion in a liquid medium. Figure 1 depicts the Block diagram of the EDM device schematically. The present experimental dielectric tank was separated by a small tank (Figure 3) and implied a 3-stage filter system ranging from 5 µm to 20 µm. The Cu workpiece was heated enough by plasma ionic shock waves to evaporate in just a few seconds by an extreme-density current pulse by EDM pulse supply system. Oleic acid was used to cool the superheated Cu particles by coating the surfaces. The heat generated by a tool and workpiece explosion stimulates the coating process between the Cu particles and the oleic acid. Variations in the applied voltage regulate the resultant particles’ size. A voltage of 6 V, 0.2 mm tool gap, and pulse rate of 100 ns with a duty cycle of 60% and 30-min machining time were used to generate the Cu particles.

The superheated Cu particles were cooled with the addition of oleic acid, which resulted in Cu particles with coated surfaces. The heat from a shock wave explosion initiated a coating reaction between oleic acid and synthesis particles. The Cu-coated oleic acid particles formed in the liquid medium were centrifuged at 10,000 rpm, washed with distilled water to remove other impurities, and finally dried.

## 3. Result and Discussion

This section summarises and discusses the main findings of the work. Scanning electron microscopy (SEM, Hitachi TM3030 Plus, Tokyo, Japan) was utilised to study the morphology and size of synthesised particles, and EDX (SwiftED 3000, from Tokyo, Japan) was utilised for element analysis. Transmission electron microscopy (TEM, FEI Tecnai G2 20 TWIN, Holland) was used to study the shape, size, and internal structure of the copper nanoparticles and revealed oleic acid coating. Fourier transforms infrared (FTIR) spectroscopy (TSN iS5, Thermo fisher scientific, Malaysia) characterised the bonding of Cu particles with oleic acid coating. X-ray diffraction analyses (Bruker D8 Advance) were used for the particles’ phase composition and crystal structure. Particles of copper coated with oleic acid were ground into powder and then compressed into pellets. The analysis of listed equipment was carried out at UMP Centre for Research in Advanced Fluid & Process.

**Figure 4:**
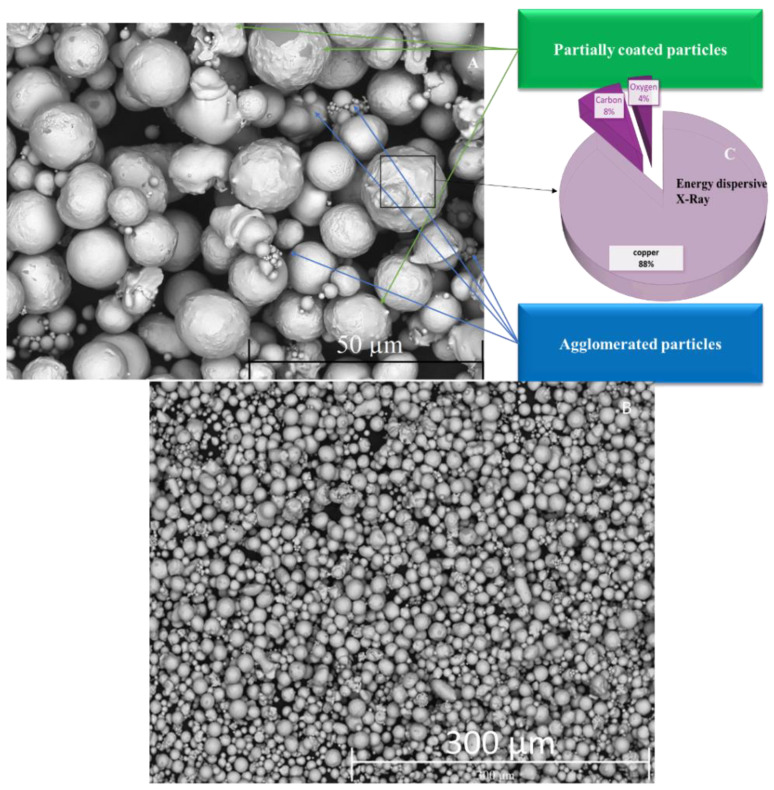
(**A**–**C**) SEM images of Synthesis particles. (**C**) represent EDX of Synthesis Particles.

**Figure 5:**
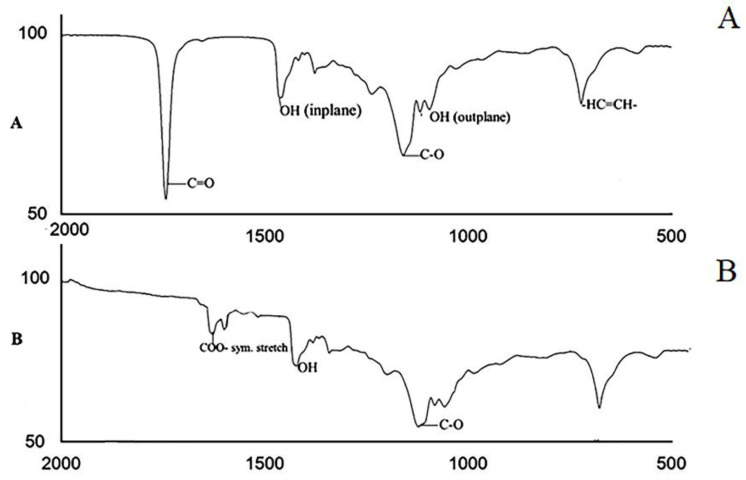
(**A**) FT-IR spectroscopy for oleic acid (C_18_H_34_O_2_); (**B**) Cu particles with oleic acid coating.

SEM images (Figure 4A,B) of copper particles show the morphology at different magnification. The SEM approach allows a more detailed analysis of some aspects of the data analysis of copper particles. After rigorous examination, it was discovered that the particle size was around 5 to 20 µm. The distribution uniformity of Cu particles with oleic acid chemically bound at their surfaces is shown in SEM micrographs, and due to excess voltage, some particles had a porous structure. The results were satisfactory, as at least in a majority of the cases the particles were spherical; however, some particles were agglomerated and non-uniform shape. The energy dispersive X-ray (EDX) describes the presence of copper 88%, carbon 8%, and oxygen 4%, as depicted in Figure 4C. Present methods have demonstrated a marked improvement in the synthesis of copper particles, and EDX demonstrated a pure form of copper. The particles coated by oleic acid-containing hexane were confirmed by FTIR analysis. FTIR spectra (Figure 5) were depicted in the 500–2000 cm^−1^ range spectra of oleic acid and Cu particles coated with oleic acid. Figure 5A shows that pure oleic acid has a strong peak at 1742 cm^−1^ due to C=O, and a band at 1238 cm^−1^ shows the presence of C-O stretch [26]. The O–H in the plane and out-of-plane bands were seen at 1463 cm^−1^ and 1095 cm^−1^, respectively, while the -HC=CH- group was attributed at 721 cm^−1^.

**Figure 6:**
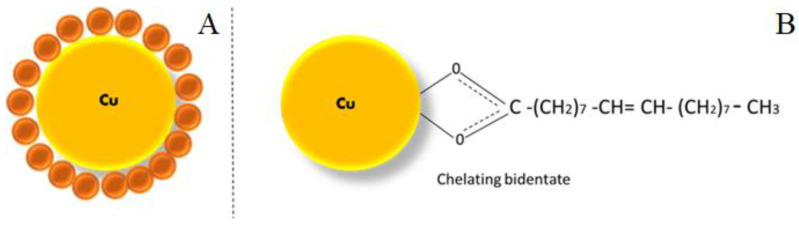
Operational prototypical of Cu particles with (**A**) oleic acid-coating. (**B**) Interface of the carboxylic group of oleic acid with copper atom.

Cu particles with oleic acid coating are shown in Figure 5B, which depicts the FTIR spectra and describes the absence of a sharp carbonyl peak stretching at 1742 cm^−1^. However, the shift to 1638 cm^−1^ and 1559 cm^−1^ are two distinct peaks representing asymmetric and symmetric (COO-) carboxylate stretching [27]. The bonding geometry in both the carboxylate head and the metal atom can be assigned to carboxylate heads and metal atoms based on the frequency difference in modes. There is covalent bonding with the COO- group and Cu atom, as evidenced by the measured splitting of about 79 cm^−1^. The vast difference in dipole moment between the -COOH polar group of oleic acid and the transition metal atom exhibits the solid electronic interaction [28]. The bonding of Cu with oleic acid is schematically represented in Figure 6.

**Figure 7:**
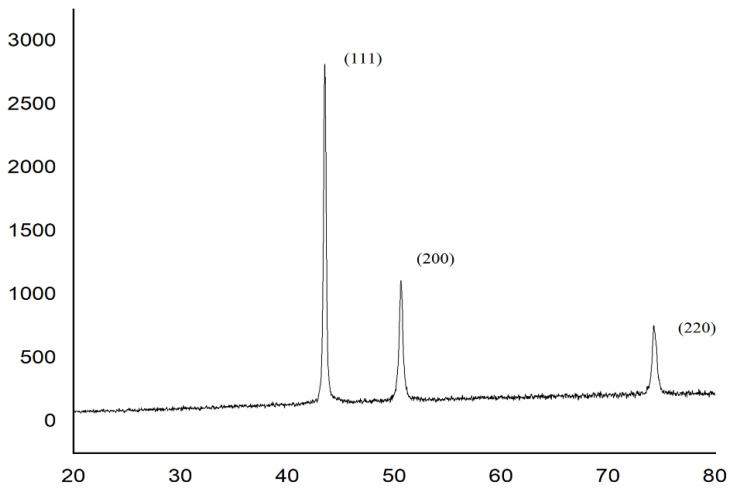
The XRD pattern of synthesis of Cu particles.

XRD observed no impurities peaks and in proper plan concerning copper as (111), (200), and (220). The XRD pattern of synthesis particles’ findings reinforces the general belief that there is no change in the shift in the crystal structure of Cu; the results of this method are in agreement with JCPDS card no 98−005−3247 [29]. The diameter of the copper particles was less than 20 µm, and they were spherical in shape. Figure 7 depicts the XRD patterns obtained from Cu particles with oleic acid coating. The corresponding planes of pure Cu were used to index all diffraction peaks. The Bragg’s reflections of the copper particles exhibited 2θ values of 43.2°, 50.4°, and 74.0°, which relate to (111), (220), and (200) [30]. It emphasised that the copper particles were free from oxidation. The oleic coating on metal particles appears to be quite effective at preventing the oxidation of metal particles in ambient air.

The results achieved surpass the earlier work in this area in particle synthesis by electrical discharge machining [8].

**Figure 8:**
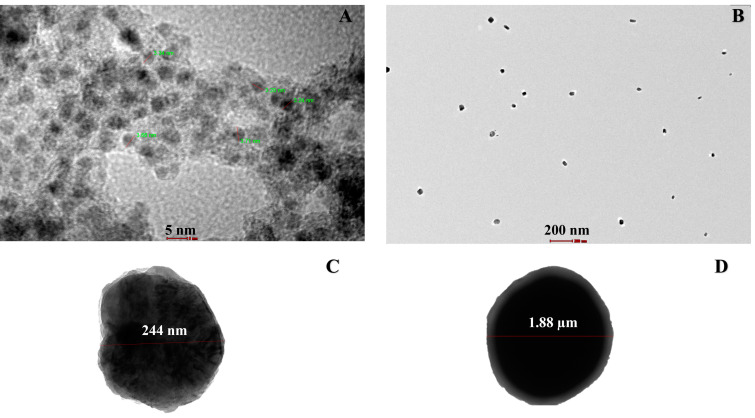
TEM images of synthesis nanoparticles at: (**A**) 280 kx; (**B**) 7.8 kx; (**C**) 62 kx; and (**D**) 6.5 kx.

The copper particle generated by the EDM machine was analysed by TEM. The results appear to make sense and be compatible with our expectations as the population of synthesised nanoparticles lies between 3 to 5 nm, and 200 nm, as shown in Figure 8A,B, respectively. In the other TEM (Figure 8C,D) particle size 244 nm and 1.88 µm was noticed. TEM images also show internal structures, revealing that particles are solid and contain metallic structures. The population of Cu nanoparticles with a diameter between 3 to 20 nanometres in conjunction with a spherical shape confirms that metal nanoparticles are in the nano range. TEM images also revealed that small particle aggregates are coated, acting as a capping agent. The white layer near-spherical area shows an oleic acid bond with copper. The present results perform in accordance to the method established by Sahu et al. [31]. They generated copper nanoparticles using micro-EDM in the range of 600 to 1100 nm using distilled water and distilled water with poly vinyl alcohol (PVA) and poly ethylene glycol (PEG) stabilising agents. However, EDX pattern shows some peaks of oxidation.

The result revealed that EDM machines could synthesise almost accurate sizes of copper particles that can use in healthcare and coating applications. However, EDM did not generate a complete controlled size, and it requires optimisation. Oleic acid has high viscosity (26.7 cP) at initial and 18 cP when machining is done; this change is due to high temperature during machining that formulates agglomeration of particles.

## 4. Conclusions

This study presents a simple approach to the synthesis of copper particles. Particles with diameters less than 20 µm were formed when an electric charge was applied to a copper workpiece in an acidic solution, and this pure Cu phase was maintained. The surface properties of various metal powders can be modified or prevented from oxidation by EDM in liquid media, which is not limited by the conducting metal, solvent, or capping agent. There must be a method to prevent the formation of aggregates between new and existing particles of copper. The usage of a stirrer in EDM processing reduces agglomeration. Due to the high voltage after thermal expansion, the particle shape changes from spherical to needle, so the exact shape of the particles can only be determined in a certain interval. Further optimisation and investigation are needed to determine whether electrical discharge machining is useful as a particle synthesis machine. However, this work suffers limitations, especially with electrically conductive materials and different voltage settings. This research work has some notable strengths, such as using waste material for industry-ready 4.0 revaluation.

## Figures and Tables

**Table 1:** Experimental condition.

Experimental Conditions	Working Parameters	Experimental Conditions	Working Parameters
Workpiece	Copper alloy	Operating voltage	230 V
Electrode	Copper	Applied voltage	6 V
Dielectric	Kerosine (layered by oleic acid)	Working time	30 min
Peak current Ip	15A	Tool gap	0.02 mm
Duty Cycle	60%	Temperature	35–40 °C

## Data Availability

The data supporting this study’s findings are available from the corresponding author upon reasonable request.
